# Global trends in surgically based treatment of anal fistula in Crohn’s disease: a bibliometric and visualization analysis

**DOI:** 10.1097/JS9.0000000000002238

**Published:** 2025-01-24

**Authors:** Xingtao Jin, Ye Han, Min Yang, Qianqian Ye, Qingming Wang, De Zheng, Zubing Mei

**Affiliations:** a Department of Anorectal Surgery, Shuguang Hospital, Shanghai University of Traditional Chinese Medicine, Shanghai, China; b Anorectal Disease Institute of Shuguang Hospital, Shanghai, China

**Keywords:** anal fistula, bibliometrics, citespace, Crohn’s disease, surgery, vOSviewer

## Abstract

**Background:**

Crohn’s disease (CD) is a chronic, recurrent gastrointestinal disorder characterized by a complex etiology. Among its perianal complications, anal fistulas represent a challenging comorbidity. With the increase of surgical options, a comprehensive bibliometric analysis was deemed necessary to consolidate the vast array of research in this field.

**Methods:**

We extracted 1608 articles spanning from 1 January 1994, to 1 May 2024, from the Web of Science Core Collection. Using VOSviewer, CiteSpace, and Scimago Graphica for visual analytics, we synthesized key trends across multiple bibliometric indicators, encompassing geographic and institutional contributions, individual authorship, journal prominence, citation metrics, and thematic prevalence.

**Results:**

From the delineated corpus, we identified publications from 325 countries and 5110 research institutions, with the US and UK at the forefront of publication volume and academic impact. The data indicated a leading role for institutions like the Cleveland Clinic and Imperial College London. “Diseases of the Colon and Rectum” emerged as a central journal due to its high publication and citation frequency. Distinctly, the analysis uncovered trending keywords, signifying the field’s prioritization on surgical intervention, biologic therapy, imaging modalities, and emerging biological treatments.

**Conclusion:**

Our findings elucidate a trajectory toward prominent advancements in CD fistula research. This analysis underscores the field’s shift towards integrative treatment strategies, spotlighting the pressing need for comprehensive comparative studies of surgical approaches. It underscores the imperative for robust clinical trials to standardize treatments and extend care to a broader CD patient population.

## Introduction

Crohn’s disease (CD) is a chronic, transmural inflammatory pathology primarily affecting the terminal ileum or colon. Its heterogeneous course is marked by alternating episodes of flare-ups and quiescence^[1]^. Within its spectrum, perianal CD manifests variably as non-fistulizing (e.g., fissures, ulcers, strictures) and fistulizing lesions, which include fistulas and abscesses. Recent insights into the pathogenesis of CD emphasize a more nuanced understanding of the interaction between genetic markers and environmental factors, particularly the gut microbiome, which may influence the severity and therapeutic response in perianal CD^[2]^. While genetic predispositions play a pivotal role, emerging research suggests that local microbiota disturbances significantly contribute to the development of complex fistulas in CD patients. This new understanding offers potential targets for novel therapeutic approaches, including microbiome modulation. Genome-wide association studies have identified more than 200 genetic polymorphisms that may contribute to the development of CD^[3]^. In a study conducted on Han Chinese individuals in South China, IRGM rs72553867, NKX2-3 rs4409764, AOX1 rs3731772, and nine SNPs located in TNFSF15 were identified as susceptibility factors for anal fistula in CD patients^[4]^. Moreover, advances in microbiological techniques have revealed a distinct microbial fingerprint associated with CD fistulas, which could be pivotal in developing personalized medicine strategies^[5]^. Researchers investigating the microbiology of perianal fistulas by aspirating and culturing pus from fistulas found that CD fistulas exhibited a preference for gram-positive bacteria over gram-negative bacteria^[6]^. Furthermore, a recent study employing a novel 16S rRNA gene sequencing technique to analyze the microbiological composition of fecal and fistula samples from patients with perianal CD revealed that fistula samples contained significantly higher levels of B. achromogenes and B. corynebacterium compared to fecal samples^[7]^. Notably, perianal fistulas are observed in 21%-23% of CD patients, more frequently associated with extensive colonic involvement than with localized small bowel disease^[8-10]^.

Globally, the morbidity associated with CD has progressively increased, with a notable incidence spike of 4%-15% annually over recent decades^[11]^. This trend is mirrored in diverse epidemiological settings – from the steady rise noted in South Korea to the prevalence upticks documented in North America^[12-14]^. Alarmingly, Crohn’s patients with perianal fistulas demonstrate a tripling in the risk of developing anorectal malignancies in contrast to the general populace^[15]^. Beyond the physiological burden, the psychosocial ramifications of CD-related fistulas, notably impinging upon social and intimate spheres of life, constitute a significant detriment to patient quality of life^[16]^.

Perianal CD, afflicting 25%-35% of the patient cohort, stands as one of the most challenging therapeutical conundrums within the disease’s clinical management. The multidisciplinary treatment arsenal spans from biologic agents and thiopurine-based therapies to combative immunosuppression and meticulous surgical interventions like abscess drainage – a crucial procedural prelude to considering definitive surgeries such as fistulotomy, flap grafting, and intersphincteric fistula ligation^[17,18]^.

The application of bibliometrics transcends mere qualitative analysis, offering a quantitative assessment of the scientific landscape. By evaluating research productivity and its influence, valuable insights can be gleaned that have far-reaching implications for clinical practice^[19,20]^. This study represents an endeavor to systematize and distill the breadth of literature concerning CD fistula research accumulated over the past thirty years. Our primary aim is to explore the evolutionary trajectory of the field, identifying prevalent research themes and potential future directions. Such intel is indispensable for clinicians seeking to align with the most up-to-date, evidence-based interventions and for researchers aspiring to plug knowledge gaps and propel the domain to new frontiers. In essence, this bibliometric analysis aspires to serve as a cornerstone in guiding future clinical and investigative pursuits tailored to optimize patient outcomes in the context of CD fistula care.

## Methods

### Date collections

**Figure 1.**
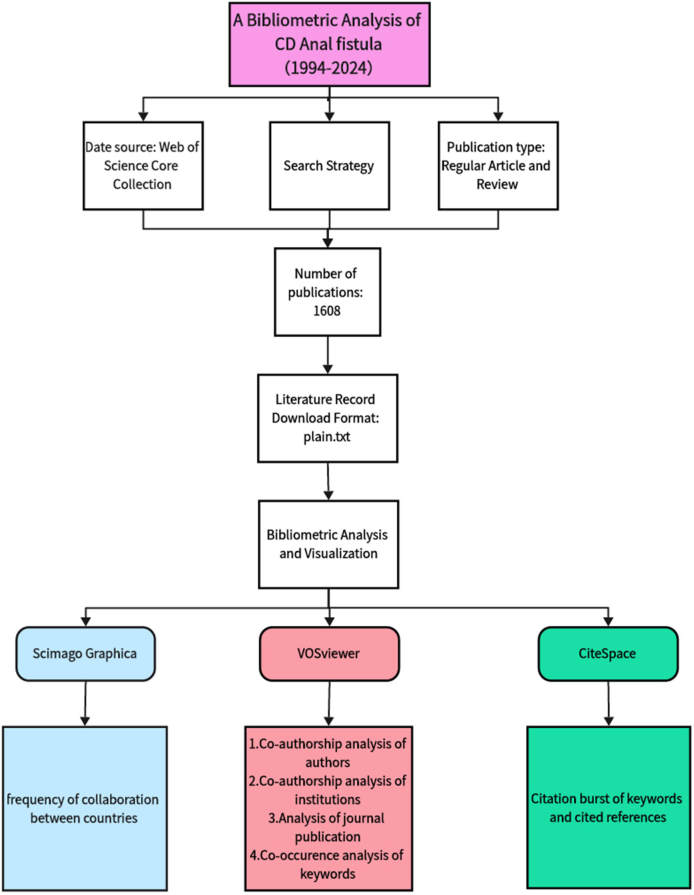
Flow-chart of the study.

We selected the Web of Science Core Collection for our study. We searched for articles on CD anal fistula published between 1 January 1994, and 1 May 2024, using the following search formula: TS = (Crohn’s disease OR CD OR Crohn disease) AND TS = (anal fistula OR fistula in ano OR perianal fistula OR anorectal fistula OR extrasphincteric fistula OR transsphincteric fistula OR intersphincteric fistula OR supralevator fistula). To facilitate further analysis, only articles and review articles were included. Complete records and cited references were then extracted from the relevant publications and saved in plain text format for subsequent analysis. The flowchart of data collection and analysis is shown in Fig. 1.

### Data extraction, standardization, and analysis

The bibliometric tools used in this article were VOSviewer (version 1.6.20), CiteSpace (version 6.3.R1), Scimago Graphica, and Microsoft Office Excel 2019. VOSviewer is a software tool for constructing and visualizing bibliometric networks. These networks may include countries, institutions, and keywords, among others, based on co-authorship, citation, co-occurrence, and bibliographic coupling data. It was specifically utilized for country, institution, and author collaborative network analysis, as well as keyword co-occurrence network analysis. CiteSpace supports the visual exploration of knowledge graphs in literature databases. It aids in the analysis of keywords and citation bursts over specific time periods, helping to identify research trends and hotspots. Scimago Graphica enhances data representation, especially in illustrating inter-country collaborations, thereby simplifying complex information for easy comparison and interpretation. Finally, Microsoft Office Excel 2019 was used to conduct the initial quantitative analyses, preparing the data for further visualization and interpretation with the aforementioned tools.

### Parameter setting

After importing the document into CiteSpace, the settings were adjusted to analyze keywords and citation nodes on a yearly basis, focusing on the top 25 most cited or mentioned items each year. We then performed an outbreak analysis after processing the document. In VOSviewer, data related to co-authorship and keyword co-occurrence were particularly targeted, setting thresholds that ensure the inclusion of the most relevant and impactful data, typically between 40 and 60 files. The results were fine-tuned and visualized to provide clear, actionable insights. Scimago Graphica was then utilized twice to map and refine these visual representations, ensuring the clarity and effectiveness of the information displayed.

The H-index, introduced by Hirsch, serves as a hybrid metric to evaluate both the quantity and quality of academic contributions made by a specific country, journal, institution, or researcher^[21]^. It is widely applicable as a relatively objective evaluation standard, helping to enhance fairness in academic assessment and to identify and incentivize high-quality research achievements. To determine the H-index for a country, all relevant articles are retrieved from the Web of Science Core Collection, then filtered by the corresponding Countries/Regions category, and a Citation Report is generated. This method can also be applied to assess the H-index of an institution. An individual researcher’s H-index can be found directly through a search by the researcher’s name, displaying their respective H-index.

## Results

### Quantitative analysis of publication

**Figure 2.**
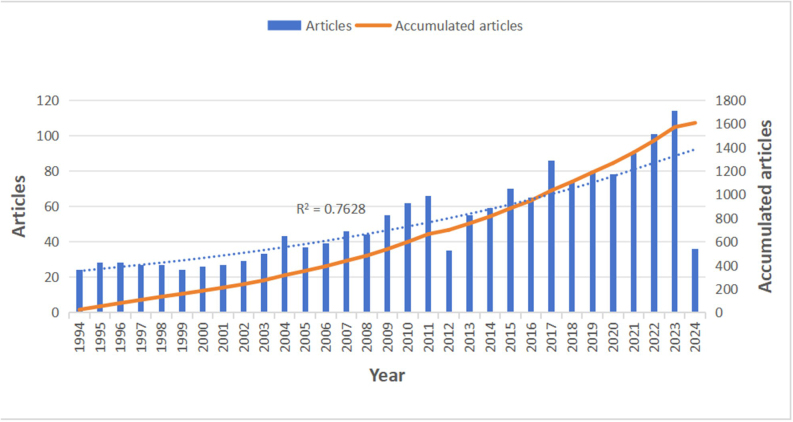
Number of publications per year and the cumulative number.

Based on the search terms, a total of 1608 articles were identified from the Web of Science Core Collection (WoSCC), including 1304 articles and 304 review articles. Fig. 2 shows the annual and cumulative number of publications related to CD anal fistula. The number of publications has steadily increased each year, from 24 in 1994 to 114 in 2023. In the last 2 years, the number of publications related to CD anal fistula has exceeded one hundred, indicating a growing interest in the topic. Specifically, 114 articles were published in 2023, the highest number among the years analyzed.

### Analysis of national publication

**Figure 3.**
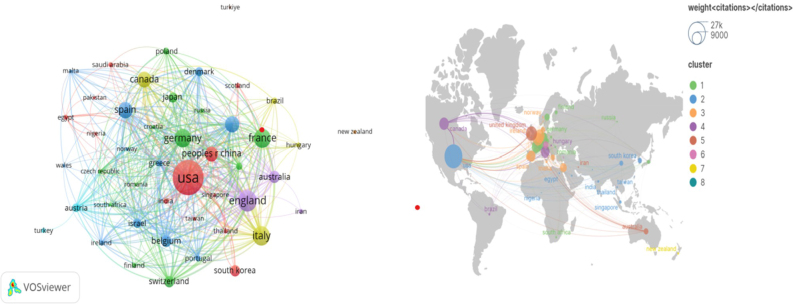
Network of cooperation in each country.

These publications came from 325 countries. Table 1 presents the top 10 countries in terms of publications, with six of them located in Europe. The top three countries accounted for more than half of the total number of publications (n = 805, 50.1%). These countries include the United States in North America (n = 478, 29.7%), the United Kingdom in Europe (n = 178, 11.1%), and Italy in Europe (n = 149, 9.3%). Using VOSviewer, countries with three or more publications were filtered and visualized. Fig. 3 displays the collaborative network among these countries based on the number and relationship of publications.

**Table 1:** Top 10 countries for publications

Rank	Country	Article	Citations	Total link strength	H-index
1	USA	478	26 483	283	79
2	England	178	10 188	284	52
3	Italy	149	6227	234	38
4	France	141	7089	141	38
5	Germany	135	5654	180	38
6	Netherlands	105	8750	192	37
7	Spain	104	5293	176	33
8	Canada	97	7453	195	34
9	Peoples Republic of China	85	1263	26	20
10	Australia	60	1806	87	19

### Analysis of institution publications

**Figure 4.**
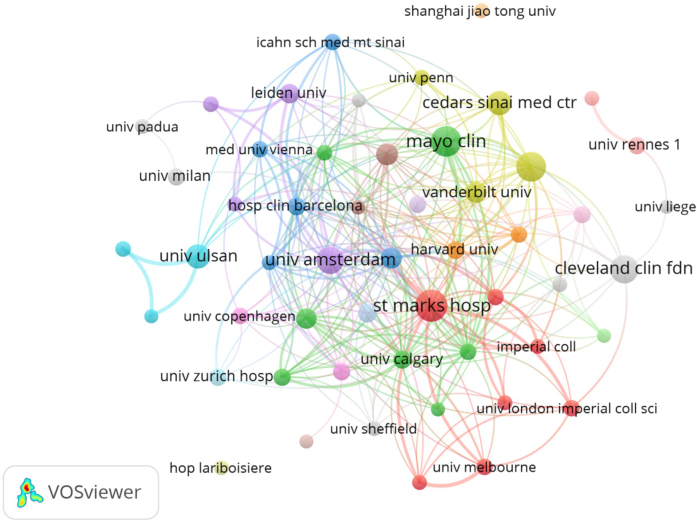
Key institutions and their relationships in this area.

Research on CD anal fistula is conducted in approximately 5110 institutions worldwide. Table 2 displays the top 10 institutions in terms of literature production. The Cleveland Clinic Foundation, an institution from the United States of America, published the highest number of articles with 103 publications, followed by Imperial College London with 79 publications from the United Kingdom. Fig. 4 illustrates the primary institutions and their collaborations in this area.

**Table 2:** Top 10 institutions for literature output

Rank	Institution	Country	Article	H-index	Total citations
1	Cleveland Clinic Foundation	USA	103	43	6487
2	Imperial College London	England	79	37	5640
3	Assistance Publique Hopitaux Paris (APHP)	France	67	26	4307
4	Universite Paris Cite	France	61	23	2502
5	Mayo Clinic	USA	58	33	5316
6	University of Amsterdam	Netherlands	52	26	5833
7	Institut National de la Sante et de la Recherche Medicale (Inserm)	France	43	18	1107
8	Harvard University	USA	39	20	4626
9	University of London	England	36	19	1456
10	University of California System	USA	35	19	1447

### Analysis of Journals

A total of 333 journals published articles on research in the field of fistula in CD. Table 3 presents the top 10 journals in order of total citations, while Table 4 displays the top 10 journals in order of the number of publications. The journal with the highest number of publications is Diseases of The Colon and Rectum, with 140 articles, which is also the most cited journal with a total of 1204 citations. Inflammatory Bowel Disease had the second highest number of articles with 110, while Gastroenterology had the second highest number of citations with 1157.

**Table 3:** The top 10 journals were ranked by total citations

Rank	Journal	Article	2023IF	JCR-c	Total citations
1	Diseases of The Colon and Rectum	140	4.1	Q1	1204
2	Gastroenterology	25	29.4	Q1	1157
3	Gut	14	24.5	Q1	1016
4	Inflammatory Bowel Disease	110	4.9	Q2	971
5	American Journal of Gastroenterology	31	10.2	Q1	890
6	British Journal of Surgery	24	9.6	Q1	799
7	Alimentary Pharmacology and Therapeutics	52	7.6	Q1	698
8	New England Journal of Medicine	2	158.5	Q1	682
9	International Journal of Colorectal Disease	42	2.8	Q2	679
10	Lancet	2	168.9	Q1	653

**Table 4:** The top 10 issues were ranked by number of publications

Rank	Journal	Article	2023IF	JCR-c	Total citations
1	Diseases of The Colon and Rectum	140	4.1	Q1	1204
2	Inflammatory Bowel Disease	110	4.9	Q2	971
3	Journal of Crohns and Colitis	72	8.0	Q1	569
4	Colorectal Disease	71	3.4	Q1	553
5	Alimentary Pharmacology and Therapeutics	52	7.6	Q1	698
6	Techniques in Coloproctology	51	3.3	Q1	311
7	International Journal of Colorectal Disease	42	2.8	Q2	679
8	World Journal of Gastroenterology	35	4.3	Q2	435
9	American Journal of Gastroenterology	31	10.2	Q1	890
10	Digestive Diseases and Sciences	27	3.1	Q3	422

### Analysis of author influence and collaboration

A total of 7645 authors are involved in the research of CD anal fistula. Table 5 presents the top 15 authors in terms of the number of publications. All the top 15 authors have published at least 15 articles. Lightner, Amy L holds the first place with 26 publications, boasting an h-index of 29, and a total number of citations of 2752. Following Lightner, Gerhard Rogler ranks second with 21 publications, an h-index of 85, and a total number of citations amounting to 46 762.

**Table 5:** The top 15 authors in the number of publications

Rank	Author	Country	Article	H-index	Total citations
1	Lightner, Amy l	USA	26	29	2752
2	Rogler, Gerhard	Swiss Confederation	21	85	46 762
3	Peyrin-Biroulet, Laurent	France	20	103	49 314
4	Shen, Bo	Peoples Republic of China	19	69	23 082
5	Siproudhis, Laurent	France	19	35	3460
6	Danese, Silvio	Italy	18	105	45 529
7	Hart, Ailsa	England	17	63	19 770
8	Schwartz, David a.	USA	17	20	998
9	Ye, Byong Duk	Korea	17	42	7324
10	Bouguen, Guillaume	France	16	34	5027
11	Fazio, Victor	USA	16	96	32 277
12	Spinelli, Antonino	Italy	16	49	9284
13	Garcia-Olmo, Damian	Spain	15	36	5879
14	Park, Sang Hyoung	Korea	15	36	4623
15	Yang, Suk-Kyun	Korea	15	50	11 798

**Figure 5.**
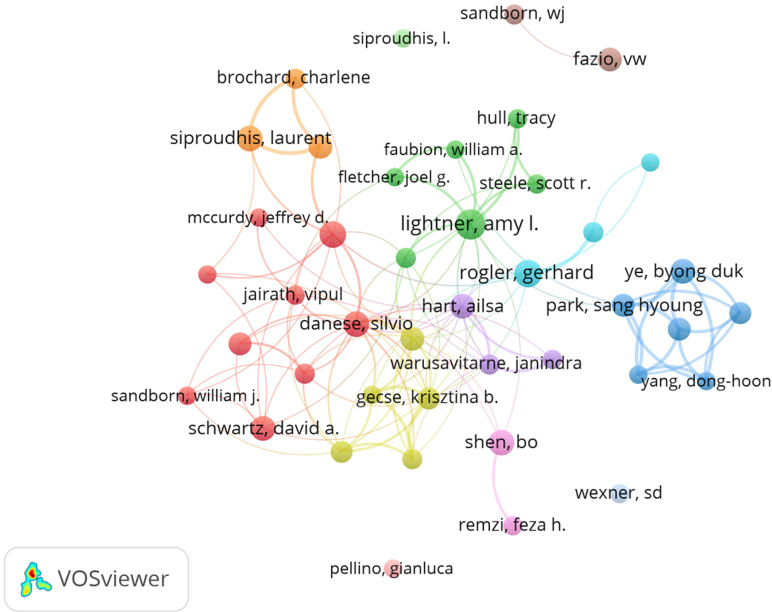
Collaborative relationships among researchers.

Furthermore, we constructed a collaborative network based on 42 authors, each with at least 10 publications. Fig. 5 illustrates the collaborations among these researchers. The size of the nodes indicates the number of authors’ postings, the color represents the time of posting, and the thickness of the connecting lines denotes the frequency of collaborations among them. It is evident that the main authors of the postings form eight primary collaborative groups.

### Co-cited references and bursts detection

Table 6 displays the 10 most cited articles, with 6 of them classified as Clinical Trials and 1 reference cited more than 100 times. The most cited article, “Expanded allogeneic adipose-derived mesenchymal stem cells (Cx601) for complex perianal fistulas in Crohn’s disease: a phase 3 randomised, double-blind controlled trial,” was published in Lancet. A citation explosion refers to a surge in citation frequency after a article is published, indicating a high level of interest in the topic.

**Table 6:** Top 10 most cited articles

Rank	Co-cited reference	Co-citation
1	Expanded allogeneic adipose-derived mesenchymal stem cells (Cx601) for complex perianal fistulas in Crohn’s disease: a phase 3 randomised, double-blind controlled trial.	126
2	Long-term Efficacy and Safety of Stem Cell Therapy (Cx601) for Complex Perianal Fistulas in Patients With Crohn’s Disease.	96
3	Infliximab for the treatment of fistulas in patients with Crohn’s disease.	65
4	Allogeneic Bone Marrow-Derived Mesenchymal Stromal Cells Promote Healing of Refractory Perianal Fistulas in Patients With Crohn’s Disease.	63
5	A global consensus on the classification, diagnosis and multidisciplinary treatment of perianal fistulising Crohn’s disease.	54
6	Infliximab maintenance therapy for fistulizing Crohn’s disease.	52
7	Modern management of perianal fistulas in Crohn’s disease: future directions.	51
8	3rd European Evidence-based Consensus on the Diagnosis and Management of Crohn’s Disease 2016: Part 2: Surgical Management and Special Situations.	50
9	Expanded allogeneic adipose-derived stem cells (eASCs) for the treatment of complex perianal fistula in Crohn’s disease: results from a multicenter phase I/IIa clinical trial.	50
10	Perianal fistulizing Crohn’s disease: pathogenesis, diagnosis and therapy.	43

**Figure 6.**
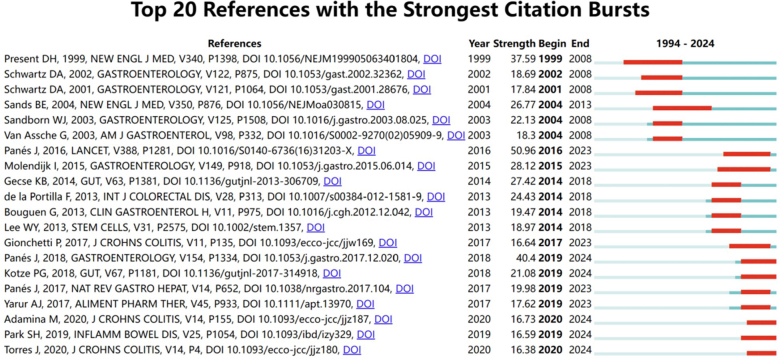
The top 20 references with the strongest citation outbreak.

Fig. 6 illustrates the top 20 references with the strongest citation bursts. The dark blue line represents the citation frequency from 1994 to 2024, while the red line indicates the range of citation bursts. The minimum burst range is 4 years. The article “Expanded allogeneic adipose-derived mesenchymal stem cells (Cx601) for complex perianal fistulas in Crohn’s disease: a phase 3 randomised, double-blind controlled trial” has the highest citation burst value from 1994 to 2024, with a strength of 50.96 and a burst period from 2016 to 2023, matching the most cited literature. The second strongest citation burst is observed in the article titled “Long-term Efficacy and Safety of Stem Cell Therapy (Cx601) for Complex Perianal Fistulas in Patients With Crohn’s Disease,” published in Gastroenterology, with a strength of 40.4 and a burst period from 2019 to 2024.

Additionally, four of these articles continue to experience citation bursts, with studies focusing on the surgical management of anal fistulas in CD.

### Keyword analysis and analysis of keywords bursts

**Figure 7.**
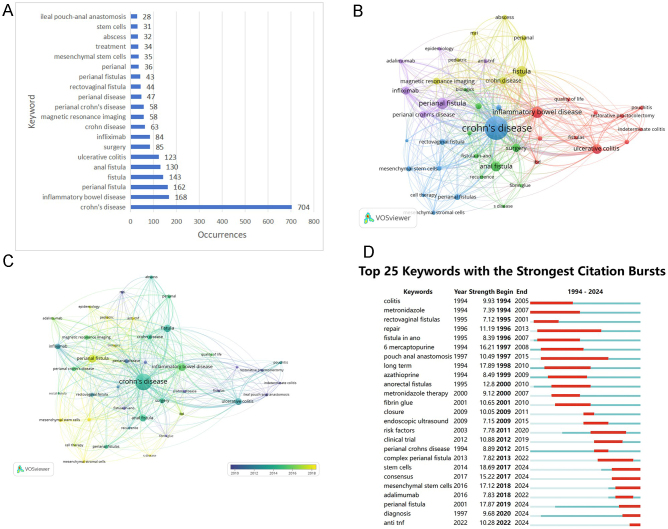
(A) A list of the 20 most frequently used keywords. (B) Keyword co-occurrence network. (C) Time-overlapping co-occurrence analysis network of keywords. (D) The top 25 keywords with the strongest citation outbreak.

Keyword co-occurrence analysis facilitated the identification of research hotspots for CD anal fistula. Fig. 7A illustrates the top 20 keywords in terms of frequency, with “Crohn’s disease” being the most frequent keyword, used 704 times. Out of the 2042 keywords found in 1608 articles, 42 keywords with a frequency of occurrence greater than or equal to 15 times were extracted and clustered. Fig. 7B presents the network visualization analysis of these keywords, where the size of the nodes reflects the keyword frequency, and the keywords are divided into five clusters. Fig. 7C visualizes the temporal overlap of keywords.

In Fig. 7D, we present the top 25 keywords with the strongest keyword bursts. The keyword “stem cells” receives the most attention, with a strength of 18.69 and a burst period from 2017 to 2024. The next most popular keyword is “long term” with a strength of 17.89 and a burst period from 1998 to 2010. More recently, keywords such as “consensus” (2017-2024), “mesenchymal stem cells” (2018-2024), “perianal fistula” (2019-2024), “diagnosis” (2020-2024), and “anti TNF” (2022-2024), among others, are still experiencing explosive usage, suggesting that future research hotspots remain focused on these keywords.

## Discussion

### Principal findings

This article analyzes the current status and trends of research on CD anal fistula using bibliometric methods. The results show that interest in CD anal fistula has increased significantly over the last 10 years, especially since 2022, marking its most productive period, with an average of more than 100 publications per year. This surge in interest may be attributed to the global rise in the incidence and prevalence of CD, with anal fistula being its main outward presenting symptom^[11]^. Consequently, research organizations have been increasing their support for anal fistula research in CD, and research funding has also been on the rise, contributing to the rapid development of this field.

A total of 325 countries participated in the research, with the top 10 countries producing 1532 articles, accounting for 95.3 percent of all publications. The United States dominates this field, with the highest publication output and a significant role in international cooperation. The fact that the top institutions and the most prolific authors are from the United States further underscores its leadership in CD fistula research.

Among the top 15 authors in terms of publications, the most prolific author is Amy L. Lightner from the Scripps Research Institute. We observe that authors from Europe and the Americas have close collaborations, whereas Asian authors, including three from Korea and one from China, mostly engage in domestic collaborations with limited international cooperation. The relative lack of research in this area in Asia, coupled with the potential for increased research competitiveness through transnational cooperation, highlights the need for broader inter-institutional collaborations, especially in contexts of economic or resource scarcity.

In terms of journal influence, impact factor and JCR (Journal Citation Reports) are effective indicators for evaluating the impact of journals. JCR Q1 journals account for 60% of the top 10 journals by number of publications. Researchers also tend to identify potential journal submissions based on the number of publications in relevant fields. Six of the top 10 journals have published more than 50 articles on CD anal fistula research. Among them, Diseases of The Colon and Rectum and Inflammatory Bowel Disease have the highest number of publications. Notably, no Asian publisher is responsible for the top 10 journals, despite China’s significant contribution to the field with a high number of articles in the top 10. This suggests that China needs to establish and develop influential journals.

The most cited article is titled “Expanded allogeneic adipose-derived mesenchymal stem cells (Cx601) for complex perianal fistulas in Crohn’s disease: a phase 3 randomised, double-blind controlled trial,” published in The Lancet. The number of citations reflects the impact of an article, and by counting highly cited articles, we can gain insight into recent research trends in the field. The two most cited articles focus on the efficacy and safety of mesenchymal stem cells (Cx601) in treating complex anal fistula in CD. The 10 most cited articles, published in the last 10 years, primarily address treatment modalities for CD fistulae, including mesenchymal stem cell therapy and infliximab therapy.

Stem cell therapy was first proposed in 1976 when Alexander Friedenstein published the pioneering research on mesenchymal stem cells (MSCs). This foundational work established the concept of MSCs and their potential in tissue regeneration and repair^[22]^. The use of MSCs in CD fistula emerged with the first publication by Garcia-Olmo *et al* in 2003, which applied MSCs to treat perianal fistula associated with CD^[23]^. This research marked an important milestone, demonstrating the effectiveness of autologous MSCs in promoting the healing of complex anal fistulas in patients with CD. The promising results of this research paved the way for further studies and clinical trials.

Stem cell therapy is emerging as a promising alternative treatment that increases local cell counts, reduces inflammation, and regenerates damaged perianal tissue. A clinical trial involving allogeneic umbilical cord MSC transplantation for Crohn’s fistula disease observed that 6 out of 10 patients achieved combined remission after 24 weeks of treatment, with a 70% probability of remaining recurrence-free at week 52^[24]^. Another phase III clinical study found that the combined remission rate using allogeneic, expanded adipose-derived stem cells was 50%^[25]^. As research on MSC treatment for CD anal fistula progresses, exosome therapy and tissue engineering – both integral parts of regenerative medicine – are gradually being explored for this condition^[26,27]^. Exosomes, recently discovered nanoscale biological vesicles, carry bioactive and signaling molecules such as proteins, lipids, and nucleic acids between cells^[28]^. While the role of exosomes in the progression of CD fistulas is not yet proven, there are reports of their involvement in inflammatory bowel disease. Some studies have even suggested the potential use of plant-derived exosome nanoparticles for treating inflammatory bowel disease^[29]^. Tissue engineering, an interdisciplinary therapeutic approach, utilizes stem cells and biomaterials in various ways to restore, maintain, and enhance the function of damaged tissues and organs^[30]^. Preliminary studies indicate that combining MSCs with biomaterials (e.g., plugs, matrices and glues) can improve the efficacy of cellular therapies in surgical procedures for patients^[31]^. Despite the significant advancements in cell therapy, exosome therapy, and tissue engineering, several challenges remain that require further research and clinical trials.

Keywords can reflect the core content of a study, and co-occurrence analysis helps identify high-frequency keywords in the same research area, indicating current hot topics. In this study, the high-frequency keywords, in addition to those that are clearly related to the search terms “Crohn’s disease” and “perianal fistula,” include “ulcerative colitis,” “surgery,” and “infliximab,” which appear most frequently. The keyword outbreak analysis also highlights some keywords that remain prominent in recent outbreaks, such as “anti-TNF,” “diagnosis,” and “perianal fistula.” Additionally, five cited references are experiencing citation bursts in 2024, primarily focusing on the treatment of CD fistula. By analyzing the explosion of keywords and references, we can identify surgical treatment of CD fistula as a potential research hotspot.

The surgical management of anal fistulas in CD has evolved significantly over time. Initially, traditional surgical techniques such as fistulotomy and seton placement were more common, aiming to open the fistula and allow for drainage. These methods often led to complications such as incontinence^[32]^. In the late 20th century, the use of setons, or hanging wires, became more common^[33]^. Setons are threads placed in the fistula to allow continuous drainage and reduce the risk of infection. This approach has improved outcomes but still has limitations, particularly in terms of cure rates and patient comfort. The introduction of biological agents, such as anti-TNF agents, has marked significant progress^[34]^. When combined with procedures such as seton placement or advancement flaps, these agents help reduce inflammation and improve surgical outcomes^[35,36]^. In recent years, regenerative medicine approaches, including the use of mesenchymal stem cells (MSCs), have shown promising results. Studies have reported healing rates of up to 80% with MSC therapy, providing a less invasive and potentially more effective alternative to traditional surgical approaches^[37,38]^. Overall, the treatment of CD anal fistulas has shifted to a multidisciplinary approach^[39,40]^, combining surgical techniques with advanced pharmacological treatments to promote healing and maintain patients’ quality of life.

The analysis of high-frequency keywords indicates that research on the use of anti-tumor necrosis factor (TNF) agents, particularly infliximab, for treating perianal fistulas in CD remains a prominent and cutting-edge focus. As a first-line therapeutic agent, infliximab (IFX) can alter the disease course of CD, promote mucosal healing, and reduce complications, hospitalizations, and surgeries. In a network meta-analysis, IFX was ranked as the top agent for inducing clinical remission among all CD patients^[41]^, and it has proven to be the most effective drug for managing perianal fistulous CD. However, a significant proportion of patients only achieve a partial response to IFX, suggesting that optimizing treatment strategies could further enhance clinical remission^[42-44]^. Consequently, current research primarily focuses on improving treatment strategies to boost remission rates. This includes efforts to establish optimal timing for IFX administration^[45]^ and exploring combination therapies to achieve remission^[46]^.

### Comparisons with previous studies

When compared to previous bibliometric analyses, our bibliometric analysis not only corroborates some of the findings of earlier works but also unveils additional dimensions, such as the upsurge in interdisciplinary research collaborations and a heightened emphasis on patient quality of life as a pivotal metric for evaluating therapeutic success^[40,47,48]^. These insights not only widen the understanding of the research landscape but also serve as critical indicators for future scholarly and clinical initiatives within the realm of CD anal fistula management.

### Strengths and limitations

The present study has several strengths. Firstly, this research presents the first bibliometric analysis of CD anal fistula using two recognized bibliometric software tools, providing insights into research trends and hotspots for future studies. Additionally, this article helps researchers quickly and conveniently identify key journals and literature in the field, enhancing their understanding of the research frontier. Furthermore, it introduces cutting-edge methods of endosurgical treatment for CD anal fistula, highlighting the trend towards multidisciplinary internal and external integration. Ultimately, this article aims to give researchers a comprehensive overview of CD anal fistula, which could contribute to improved clinical prognostic management and reduce its associated mortality.

This research acknowledges several limitations. Firstly, as a bibliometric analysis, the collection and processing of data rely heavily on software tools. While this approach cannot entirely replace systematic searching, it enables comprehensive analyses of large datasets. Secondly, this research only collected literature from WoSCC database, which is a common limitation in bibliometric analyses. This means some valuable studies from other databases may have been missed. However, WoSCC offers broader literature coverage than databases such as Scopus, Medline, and PubMed, suggesting that this limitation may not significantly affect the overall trends identified. Finally, the impact of recently published high-quality studies may not be fully recognized due to citation impact delays. This aspect warrants further attention in future studies and timely updates to reflect new findings.

### Clinical implications for clinicians and policy-makers

The clinical ramifications of our bibliometric analysis extend beyond academic discourse into tangible strategies for healthcare delivery. The elucidation of trends in CD anal fistula research underscores the pertinent need for a paradigm shift in therapeutic approaches, embracing an array of innovative endosurgical techniques that have emerged over the trailblazing thirty-year span. Notably, our findings advocate for a transition toward integrated, multidisciplinary treatment modalities, which resonate with the increasingly complex tapestry of CD manifestations encountered in clinical settings.

For practitioners, the emphasis on multidisciplinary strategies translates into a holistic patient management ethos, where gastroenterologists, colorectal surgeons, radiologists, and immunologists synergize their expertise. This collaboration manifests as a concerted effort encompassing accurate diagnostic imaging, optimized pharmacotherapy, and individualized surgical interventions. Given the dynamic landscape of therapeutic options, continuous professional development becomes imperative, maintaining clinical competency with the relentless pace of innovation.

Policy makers, on the other hand, are positioned to leverage these research insights by tailoring health policy frameworks that reflect the evolving standards of care. Allocating resources toward enhancing access to new biologic agents, advanced imaging modalities, and specialized surgical services could markedly improve patient outcomes. In concert with resource allocation, fostering research is pivotal; thus, policies might be reoriented to encourage trials exploring the comparative efficacy of different treatment regimes, thereby refining clinical guidelines.

Our research serves as a compendium for these stakeholders, offering a prism through which the trajectory of CD anal fistula management can be perceived. This engagement with current research paradigms not only portends the alleviation of disease burden but also facilitates a patient-centric, outcome-oriented healthcare system. It invites a reflection on current practice patterns, encouraging clinicians and policymakers alike to harmonize their endeavors with the latest evidence-based advancements for bolstering patient care quality for those afflicted by CD.

## Conclusion

In summary, the research of anal fistula in CD is receiving increasing attention, as evidenced by the annual rise in published literature, highlighting the growing importance of this research area. This research illustrates the development and changes in the field over the past decades through bibliometric and visualization techniques. The trends suggest that future research hotspots will focus on advances in the treatment of CD anal fistula, including emerging endosurgical treatments such as stem cell therapy and anti-tumor necrosis factor agents. By analyzing these trends, researchers can prepare for further investigations, and it is hoped that the resulting findings will help clinics better manage patients with CD anal fistula, leading to improved prognostic outcomes.

## Data Availability

The data in this study is not sensitive in nature and is accessible in the public domain. The data is therefore available and not of a confidential nature.

## References

[1] Gomollón F Dignass A Annese V. 3rd European evidence-based consensus on the diagnosis and management of Crohn’s disease 2016: part 1: diagnosis and medical management. J Crohns Colitis 2017;11:3–25. 27660341 10.1093/ecco-jcc/jjw168

[2] Cao S Colonna M Deepak P. Pathogenesis of perianal fistulising Crohn’s disease: current knowledge, gaps in understanding, and future research directions. J Crohns Colitis 2023;17:1010–22. 36655753 10.1093/ecco-jcc/jjad008

[3] Verstockt B Smith KG Lee JC. Genome-wide association studies in Crohn’s disease: past, present and future. Clin Transl Immunology 2018;7:e1001. 29484179 10.1002/cti2.1001 PMC5822399

[4] Zhang M Wang X Jiang X. Polymorphisms of the TNF gene and three susceptibility loci are associated with Crohn’s disease and perianal fistula Crohn’s disease: a study among the Han population from South China. Med Sci Monit 2019;25:9637–50. 31844038 10.12659/MSM.917244 PMC6929548

[5] Breton J Tanes C Tu V. A microbial signature for paediatric perianal Crohn’s disease. J Crohns Colitis 2022;16:1281–92. 35211723 10.1093/ecco-jcc/jjac032

[6] West RL Van Der Woude CJ Endtz HP. Perianal fistulas in Crohn’s disease are predominantly colonized by skin flora: implications for antibiotic treatment? Dig Dis Sci 2005;50:1260–63. 16047469 10.1007/s10620-005-2769-4

[7] Haac BE Palmateer NC Seaton ME. A distinct gut microbiota exists within Crohn’s disease-related perianal fistulae. J Surg Res 2019;242:118–28. 31075656 10.1016/j.jss.2019.04.032

[8] Van Assche G Dignass A Panes J. The second European evidence-based Consensus on the diagnosis and management of Crohn’s disease: special situations. J Crohns Colitis 2010;4:63–101. 21122490 10.1016/j.crohns.2009.09.009

[9] Ingle SB Loftus EV Jr. The natural history of perianal Crohn’s disease. Dig Liver Dis 2007;39:963–69. 17720635 10.1016/j.dld.2007.07.154

[10] Juncadella AC Alame AM Sands LR. Perianal Crohn’s disease: a review. Postgrad Med 2015;127:266–72. 25746229 10.1080/00325481.2015.1023160

[11] Ng SC Shi HY Hamidi N. Worldwide incidence and prevalence of inflammatory bowel disease in the 21st century: a systematic review of population-based studies. Lancet 2017;390:2769–78. 29050646 10.1016/S0140-6736(17)32448-0

[12] Park SH Kim Y-J Rhee KH. A 30-year trend analysis in the epidemiology of inflammatory bowel disease in the Songpa-Kangdong District of Seoul, Korea in 1986–2015. J Crohns Colitis 2019;13:1410–17. 30989166 10.1093/ecco-jcc/jjz081

[13] Zheng JJ Zhu XS Huangfu Z. Prevalence and incidence rates of Crohn’s disease in mainland China: a meta-analysis of 55 years of research. J Dig Dis 2010;11:161–66. 20579219 10.1111/j.1751-2980.2010.00431.x

[14] Benchimol EI Manuel DG Guttmann A. Changing age demographics of inflammatory bowel disease in Ontario, Canada: a population-based cohort study of epidemiology trends. Inflamm Bowel Dis 2014;20:1761–69. 25159453 10.1097/MIB.0000000000000103

[15] El-Hussuna A Lemser CE Iversen AT. Risk of anorectal cancer in patients with Crohn’s disease and perianal fistula: a nationwide Danish cohort study. Colorectal Dis 2023;25:1453–59. 37086006 10.1111/codi.16581

[16] Mahadev S Young JM Selby W. Self-reported depressive symptoms and suicidal feelings in perianal Crohn’s disease. Colorectal Dis 2012;14:331–35. 21689304 10.1111/j.1463-1318.2011.02613.x

[17] Adamina M Bonovas S Raine T. ECCO guidelines on therapeutics in Crohn’s disease: surgical treatment. J Crohns Colitis 2020;14:155–68. 31742338 10.1093/ecco-jcc/jjz187

[18] Meima-van Praag EM Buskens CJ Hompes R. Surgical management of Crohn’s disease: a state of the art review. Int J Colorectal Dis 2021;36:1133–45. 33528750 10.1007/s00384-021-03857-2 PMC8119249

[19] Jiang D Ji T Liu W. Four decades of clinical liver transplantation research: results of a comprehensive bibliometric analysis. Transplantation 2022;106:1897–908. 35831925 10.1097/TP.0000000000004224

[20] He X Xu S Tang L. Insights into the history and tendency of liver transplantation for liver cancer: a bibliometric-based visual analysis. Int J Surg 2024;110:406–18. 37800536 10.1097/JS9.0000000000000806 PMC10793788

[21] Ioannidis JPA Baas J Klavans R. A standardized citation metrics author database annotated for scientific field. PLoS Biol 2019;17:e3000384. 31404057 10.1371/journal.pbio.3000384 PMC6699798

[22] Friedenstein AJ. Precursor cells of mechanocytes. Int Rev Cytol 1976;47:327–59. 11195 10.1016/s0074-7696(08)60092-3

[23] García-Olmo D García-Arranz M Gómez García L. Autologous stem cell transplantation for treatment of rectovaginal fistula in perianal Crohn’s disease: a new cell-based therapy. Int J Colorectal Dis 2003;18:451–54. 12756590 10.1007/s00384-003-0490-3

[24] Wei J Zhang Y Chen C. Efficacy and safety of allogeneic umbilical cord-derived mesenchymal stem cells for the treatment of complex perianal fistula in Crohn’s disease: a pilot study. Stem Cell Res Ther 2023;14:311. 37904247 10.1186/s13287-023-03531-0 PMC10617053

[25] Panés J García-Olmo D Van Assche G. Expanded allogeneic adipose-derived mesenchymal stem cells (Cx601) for complex perianal fistulas in Crohn’s disease: a phase 3 randomised, double-blind controlled trial. Lancet 2016;388:1281–90. 27477896 10.1016/S0140-6736(16)31203-X

[26] Nazari H Naei VY Tabasi AH. Advanced regenerative medicine strategies for treatment of perianal fistula in Crohn’s disease. Inflamm Bowel Dis 2022;28:133–42. 34291798 10.1093/ibd/izab151

[27] Geldof J Iqbal N LeBlanc J-F. Classifying perianal fistulising Crohn’s disease: an expert consensus to guide decision-making in daily practice and clinical trials. Lancet Gastroenterol Hepatol 2022;7:576–84. 35325623 10.1016/S2468-1253(22)00007-3

[28] Mitsuhashi S Feldbrügge L Csizmadia E. Luminal extracellular vesicles (EVs) in inflammatory bowel disease (IBD) exhibit proinflammatory effects on epithelial cells and macrophages. Inflamm Bowel Dis 2016;22:1587–95. 27271497 10.1097/MIB.0000000000000840 PMC4911338

[29] Li D-F Tang Q Yang M-F. Plant-derived exosomal nanoparticles: potential therapeutic for inflammatory bowel disease. Nanoscale Adv 2023;5:3575–88. 37441251 10.1039/d3na00093a PMC10334410

[30] Nam SY Park SH. ECM based bioink for tissue mimetic 3D bioprinting. Adv Exp Med Biol 2018;1064:335–53. 30471042 10.1007/978-981-13-0445-3_20

[31] Stamos MJ Snyder M Robb BW. Prospective multicenter study of a synthetic bioabsorbable anal fistula plug to treat cryptoglandular transsphincteric anal fistulas. Dis Colon Rectum 2015;58:344–51. 25664714 10.1097/DCR.0000000000000288

[32] Tozer PJ Burling D Gupta A. Review article: medical, surgical and radiological management of perianal Crohn’s fistulas. Aliment Pharmacol Ther 2011;33:5–22. 21083581 10.1111/j.1365-2036.2010.04486.x

[33] Mujukian A Zaghiyan K Banayan E. Outcomes of definitive draining seton placement for complex anal fistula in Crohn’s disease. Am Surg 2020;86:1368–72. 33079581 10.1177/0003134820964462

[34] Meima-van Praag EM van Rijn KL Wasmann KATGM. Short-term anti-TNF therapy with surgical closure versus anti-TNF therapy in the treatment of perianal fistulas in Crohn’s disease (PISA-II): a patient preference randomised trial. Lancet Gastroenterol Hepatol 2022;7:617–26. 35427495 10.1016/S2468-1253(22)00088-7

[35] Haennig A Staumont G Lepage B. The results of seton drainage combined with anti-TNFα therapy for anal fistula in Crohn’s disease. Colorectal Dis 2015;17:311–19. 25425534 10.1111/codi.12851

[36] El-Gazzaz G Hull T Church JM. Biological immunomodulators improve the healing rate in surgically treated perianal Crohn’s fistulas. Colorectal Dis 2012;14:1217–23. 22251452 10.1111/j.1463-1318.2012.02944.x

[37] Garcia-Olmo D Gilaberte I Binek M. Follow-up study to evaluate the long-term safety and efficacy of darvadstrocel (mesenchymal stem cell treatment) in patients with perianal fistulizing Crohn’s disease: ADMIRE-CD phase 3 randomized controlled trial. Dis Colon Rectum 2022;65:713–20. 34890373 10.1097/DCR.0000000000002325 PMC8985696

[38] Wang H Jiang HY Zhang YX. Mesenchymal stem cells transplantation for perianal fistulas: a systematic review and meta-analysis of clinical trials. Stem Cell Res Ther 2023;14:103. 37101285 10.1186/s13287-023-03331-6 PMC10134595

[39] Abushamma S Ballard DH Smith RK. Multidisciplinary management of perianal Crohn’s disease. Curr Opin Gastroenterol 2021;37:295–305. 33899777 10.1097/MOG.0000000000000751

[40] Lightner AL Faubion WA Fletcher JG. Interdisciplinary management of perianal Crohn’s disease. Gastroenterol Clin North Am 2017;46:547–62. 28838414 10.1016/j.gtc.2017.05.008

[41] Barberio B Gracie DJ Black CJ. Efficacy of biological therapies and small molecules in induction and maintenance of remission in luminal Crohn’s disease: systematic review and network meta-analysis. Gut 2023;72:264–74. 35907636 10.1136/gutjnl-2022-328052

[42] Sandborn WJ. Preventing antibodies to infliximab in patients with Crohn’s disease: optimize not immunize. Gastroenterology 2003;124:1140–45. 12671907 10.1053/gast.2003.50182

[43] Gecse KB Végh Z Lakatos PL. Optimizing biological therapy in Crohn’s disease. Expert Rev Gastroenterol Hepatol 2016;10:37–45. 26471077 10.1586/17474124.2016.1096198

[44] Vasudevan A Ip F Liew D. The cost-effectiveness of initial immunomodulators or infliximab using modern optimization strategies for Crohn’s disease in the biosimilar era. Inflamm Bowel Dis 2020;26:369–79. 31532479 10.1093/ibd/izz159

[45] Sun X-L Chen S-Y Tao-S-S. Optimized timing of using infliximab in perianal fistulizing Crohn’s disease. World J Gastroenterol 2020;26:1554–63. 32327905 10.3748/wjg.v26.i14.1554 PMC7167413

[46] Luber RP Dawson L Munari S. Thiopurines and their optimization during infliximab induction and maintenance: a retrospective study in Crohn’s disease. J Gastroenterol Hepatol 2021;36:990–98. 32881046 10.1111/jgh.15245

[47] Adegbola SO Dibley L Sahnan K. Burden of disease and adaptation to life in patients with Crohn’s perianal fistula: a qualitative exploration. Health Qual Life Outcomes 2020;18:370. 33218361 10.1186/s12955-020-01622-7 PMC7678264

[48] Karki C Athavale A Abilash V. Multi-national observational study to assess quality of life and treatment preferences in patients with Crohn’s perianal fistulas. World J Gastrointest Surg 2023;15:2537–52. 38111766 10.4240/wjgs.v15.i11.2537 PMC10725550

